# Minimally invasive versus open surgery in uterine serous carcinoma: impact on recurrence and survival in a multicenter cohort

**DOI:** 10.3389/fonc.2025.1665803

**Published:** 2025-10-10

**Authors:** Yi Fang, Jian Chen, Lin Yang, Yingtao Lin, Yao Lin, Rong Lin, Xingfa Chen

**Affiliations:** ^1^ Department of Gynecology, Clinical Oncology School of Fujian Medical University, Fujian Cancer Hospital, Fuzhou, China; ^2^ Department of Clinical Medical Research Center, Clinical Oncology School of Fujian Medical University, Fujian Cancer Hospital, Fuzhou, China; ^3^ Department of Obstetrics and Gynecology, The First Hospital Affiliated to Fujian Medical University, Fuzhou, China; ^4^ Department of Obstetrics and Gynecology, Fujian Provincial Hospital, Fuzhou, China; ^5^ Department of Radiology, Clinical Oncology School of Fujian Medical University, Fujian Cancer Hospital, Fuzhou, China

**Keywords:** uterine serous carcinoma, minimally invasive surgery, open surgery, progression-free survival, overall survival, surgical outcomes

## Abstract

**Background:**

Uterine serous carcinoma (USC) is a highly aggressive subtype of endometrial cancer, characterized by high recurrence rates and poor prognosis. While minimally invasive surgery (MIS) is commonly used in endometrial cancer treatment, its oncologic safety in high-risk USC remains unclear. This study aimed to compare survival outcomes between MIS and open surgery in patients with USC.

**Methods:**

In this multicenter retrospective cohort study, 176 patients with USC who underwent primary surgical treatment were included (MIS: 53 [30.1%], open: 123 [69.9%]). Kaplan–Meier analysis was used to estimate overall survival (OS) and progression-free survival (PFS), while Cox regression identified independent prognostic factors.

**Results:**

The median follow-up was 78 months (95% CI: 68.3–87.7). Patients in the MIS group experienced a higher recurrence rate (49.1% vs. 31.7%) and lower 5-year PFS (49.7% vs. 68.3%, P = 0.017), although 5-year OS was comparable between groups (69.7% vs. 77.4%, P = 0.219). Multivariate analysis confirmed that MIS as an independent predictor of poorer PFS (HR = 2.29, 95% CI: 1.31–4.01, P = 0.004). In contrast, adjuvant therapy significantly improved PFS (HR = 0.28, 95% CI: 0.13–0.60, P = 0.001). Hypertension was also associated with decreased OS (HR = 2.06, 95% CI: 1.11–3.81, P = 0.022).

**Conclusions:**

MIS may be associated with an increased risk of recurrence and reduced PFS in USC patients, while adjuvant therapy remains critical for improving survival outcomes.

## Introduction

Although uterine serous carcinoma (USC) comprises just 5–10% of endometrial cancers, it disproportionately contributes to mortality, accounting for nearly 40% of related deaths due to its aggressive nature (1). Unlike endometrioid adenocarcinoma, USC is a non–estrogen-dependent (Type II) tumor characterized by frequent TP53 mutations, marked genomic instability, and a tendency for extrauterine dissemination even at early stages (2). Consequently, USC exhibits a high recurrence rate ranging from 31% to 80%, and its five-year overall survival (OS) rate remains below 30% (3).

The standard initial management for USC includes comprehensive surgical staging—such as hysterectomy, bilateral salpingo-oophorectomy, lymphadenectomy, and omentectomy—followed by adjuvant chemotherapy and/or radiotherapy based on postoperative pathology (4). Laparoscopic and robotic-assisted minimally invasive surgery (MIS) has increasingly been adopted as the preferred method for treating endometrial cancer. Randomized controlled trials (RCTs) have demonstrated that compared to open surgery, MIS leads to fewer postoperative complications, shorter hospital stays, and better quality of life, with no significant difference in OS or progression-free survival (PFS) (5–7). However, these studies primarily included patients with low-risk endometrioid adenocarcinoma, providing limited data on high-risk subtypes such as USC. For instance, in an RCT comparing laparoscopic and open surgery for stage I endometrial cancer, only 2.5% of the 760 participants had USC (5). Similarly, in the LAP-2 trial, only 289 of 2,616 patients (11%) had USC (7). Furthermore, these studies did not stratify outcomes by histologic subtype and mostly included early-stage cases. As a result, data on the surgical and adjuvant treatment outcomes for USC remain scarce, underscoring the need for further research.

Evidence specific to high-risk endometrial cancer indicates that MIS does not compromise oncologic outcomes when comprehensive staging is performed. In uterine serous carcinoma (USC), a large single-center cohort (n=391) demonstrated similar 5-year PFS and OS with MIS versus laparotomy after adjustment for stage and pathologic factors (8). In broader high-risk histologies (serous, clear cell, grade 3 endometrioid, carcinosarcoma), a multicenter cohort and two systematic reviews/meta-analyses found no significant differences in disease-free or overall survival between MIS and open approaches (9–11). Consistently, for uterine clear cell carcinoma, a multicenter series reported oncologic equivalence of laparoscopy versus laparotomy (12). Although USC has a predilection for multiquadrant peritoneal dissemination, careful omental assessment during MIS minimizes the risk of understaging (13).

Notably, the LACC trial challenged prior assumptions about the oncologic safety of minimally invasive radical hysterectomy, reporting significantly poorer disease-free survival (DFS) and OS outcomes in patients with early-stage cervical cancer undergoing MIS compared to those undergoing open surgery (14, 15). The investigators attributed the inferior outcomes to factors such as the use of uterine manipulators and carbon dioxide pneumoperitoneum, which may contribute to tumor spillage and peritoneal dissemination. These mechanisms are not unique to cervical cancer and are also present during MIS for endometrial cancer. Consequently, these unexpected findings have sparked ongoing debate and raised concerns regarding the applicability of MIS in high-risk endometrial cancer subtypes such as USC. Against this backdrop, the present study sought to evaluate the long-term survival outcomes of minimally invasive versus open surgery in USC patients and to explore clinical determinants associated with prognosis. In this multicenter retrospective cohort study, the co-primary outcomes were PFS and OS. Secondary outcomes included recurrence patterns by surgical approach and the identification of independent prognostic factors for PFS and OS.

## Materials and methods

### Design and patient cohort

Between January 1, 2007, and December 31, 2022, data from patients diagnosed with USC and treated at three tertiary cancer centers were retrospectively collected for this multicenter cohort analysis. Institutional Review Board (IRB) approval was obtained from the Ethics Committee of Clinical Oncology School of Fujian Medical University, Fujian Cancer Hospital (Approval No. K2025-118-01; approved on 24/03/2025). Patient data were accessed between 20/04/2025 and 31/05/2025. Given the retrospective nature of the study, written informed consent was waived for the majority of patients whose anonymized clinical data were already available in the hospital information system, in accordance with an IRB-approved waiver granted on 25/04/2025. For a small subset of patients whose survival status was incomplete and required telephone follow-up, verbal informed consent was obtained prior to contact, as approved in the original ethics protocol. These telephone follow-ups were conducted between 01/05/2025 and 11/05/2025, in accordance with the IRB approval.

Eligible patients met the following criteria: (1) histologically confirmed USC, defined as cases in which the serous component constituted ≥20% of the tumor on hematoxylin–eosin sections, consistent with thresholds used in prior multi-institutional USC cohorts evaluating HER2 and adjuvant therapy (16, 17), (2) aged between 18 and 80 years, (3) receipt of definitive surgery comprising at minimum a hysterectomy and bilateral salpingo-oophorectomy, and (4) complete postoperative follow-up information available by June 20, 2024. Patients were excluded for any of the following: serous component <20%; prior or concurrent invasive malignancy within 5 years; lack of minimum surgical staging (no hysterectomy with bilateral salpingo-oophorectomy); or absent outcome follow-up information.

### Clinical management and treatment protocols

Preoperative evaluation routinely included pelvic magnetic resonance imaging and contrast-enhanced chest–abdominal–pelvic computed tomography; positron emission tomography–CT was obtained at the investigator’s discretion when extrauterine disease was suspected. Definitive management followed FIGO criteria. Early-stage disease (FIGO I–II) underwent comprehensive surgical staging—total hysterectomy with bilateral salpingo-oophorectomy, pelvic lymphadenectomy with or without para-aortic lymphadenectomy, and infracolic omentectomy or omental biopsy—with meticulous inspection of peritoneal surfaces (including diaphragm, peritoneum, and bowel) and biopsy of all suspicious lesions. Advanced-stage disease (FIGO III–IV) underwent cytoreductive surgery. Neoadjuvant chemotherapy (NACT) was allowed for clinically advanced or bulky disease with interval cytoreduction when feasible; otherwise, patients underwent primary surgery followed by adjuvant therapy as indicated. The surgical approach (minimally invasive or open) was selected by the gynecologic oncologist according to patient factors and tumor extent. Surgical approach was classified *a priori* as minimally invasive surgery (MIS; conventional laparoscopy or robotic-assisted laparoscopy) or open surgery (laparotomy); laparoscopic and robotic procedures were pooled as MIS for the main analyses.

Adjuvant treatment followed institutional protocols in line with international guidelines: chemotherapy was taxane–platinum based, typically paclitaxel plus a platinum agent administered every three weeks for 4–6 planned cycles, with dose or cycle modifications according to tolerance; radiotherapy comprised pelvic external-beam irradiation (45–50.4 Gy in 1.8–2.0 Gy fractions) with an optional high-dose-rate vaginal brachytherapy boost at the discretion of the treating radiation oncologist. Adjuvant therapy was delivered either at the index centers or, frequently in our regional hub-and-spoke model, at patients’ local hospitals after discharge; for off-site care, multidisciplinary clearance and scheduling were completed in the outpatient setting, and the surgery-to-adjuvant interval was recorded as defined. The interval from surgery to the initiation of adjuvant therapy was prespecified and abstracted from the medical record as the number of days from the date of definitive surgery to the first chemotherapy administration or the first radiotherapy fraction.

Adjuvant therapy was initiated as soon as medically feasible following postoperative recovery. Adjuvant radiotherapy was commenced once the vaginal cuff had healed and no later than 12 weeks after surgery, whereas adjuvant chemotherapy was typically started within approximately 3–6 weeks postoperatively, with the exact timing individualized according to recovery status and the absence of complications. To quantify timing, the interval from surgery to adjuvant initiation was abstracted from the medical record as the number of days from the date of definitive surgery to the first chemotherapy infusion or the first radiotherapy fraction.

### Data collection and outcomes

OS and PFS served as the primary endpoints of this study. OS was measured from the surgical intervention to death from any cause or final follow-up. PFS represented the duration from surgery to either disease progression—confirmed by imaging or histopathology—or death, whichever occurred first. The secondary objective was to explore independent predictors of OS and PFS. First-site recurrence was evaluated at the time of initial failure. Because synchronous failures may involve multiple sites, we applied a multiple-counting approach, whereby each patient with multi-site recurrence contributed one count to every affected site; thus, site-specific proportions could exceed 100%. Post-recurrence treatments—including chemotherapy, radiotherapy (brachytherapy/IMRT), salvage surgery, targeted therapy, and immunotherapy—were extracted from medical records and summarized descriptively, with categories not mutually exclusive. For “% of recurred,” the denominator was the number of patients who experienced recurrence (N = 65).

Data collection included demographic characteristics (e.g., age at diagnosis), comorbidities (e.g., hypertension, diabetes, obesity), laboratory markers (e.g., D-dimer, fibrinogen), tumor biological parameters (e.g., International Federation of Gynecology and Obstetrics (FIGO) stage, cervical stromal involvement, lymph node metastasis), treatment characteristics (e.g., surgical approach, adjuvant therapy regimen) and follow-up data (e.g., recurrence patterns, survival status). Disease stage was classified based on the 2009 FIGO staging criteria for endometrial cancer. Adjuvant therapy included adjuvant chemotherapy and/or adjuvant radiotherapy. To comprehensively assess treatment characteristics, adjuvant therapy data were categorized into adjuvant radiotherapy (including retroperitoneal radiotherapy and brachytherapy) and adjuvant chemotherapy. Radiotherapy-related variables included the proportion of patients receiving radiotherapy, retroperitoneal radiation, and brachytherapy, as well as the median vaginal radiation dose (cGy) and the time from surgery to radiotherapy initiation. Chemotherapy-related variables encompassed the proportion of patients receiving chemotherapy, the interval between surgery and chemotherapy initiation, and the number of chemotherapy cycles.

### Statistical analysis

Prior to analysis, data completeness was reviewed across outcomes and covariates; no statistical imputation was performed. Kaplan–Meier analyses included all patients with available survival follow-up. For multivariable Cox models, a complete-case approach was applied—only participants with non-missing values for all covariates included in a given model were analyzed—and the effective sample size therefore may differ across models. To preserve power and avoid instability due to selective missingness, covariates with substantial missingness (notably CA125 level and MMR status) were not entered into multivariable models.

All analyses were conducted using SPSS software version 24.0 (IBM Corp., Armonk, NY). Continuous and categorical variables were summarized as mean ± SD or median (IQR). Appropriate parametric or nonparametric tests were applied to compare baseline characteristics across groups. OS and PFS were estimated via Kaplan–Meier curves, with differences assessed using log-rank tests. Multivariable Cox models were constructed using a two-step approach: variables with P < 0.05 in univariable analyses were entered, together with a prespecified set of clinically relevant covariates based on established prognostic factors and data availability (FIGO stage, depth of myometrial invasion, lymphovascular space invasion, cervical stromal involvement, nodal status, adnexal/omental involvement, surgical approach, and adjuvant therapy), which were retained irrespective of univariable P values.

## Results

### Clinical and pathological characteristics

A total of 197 patients with pathologically confirmed USC were retrospectively identified across three tertiary cancer centers in China: Fujian Cancer Hospital, Fujian Provincial Hospital, and the First Affiliated Hospital of Fujian Medical University. The study period spanned from January 1, 2007, to December 31, 2022. After screening, 21 patients were excluded for the following reasons: eight had serous carcinoma components accounting for less than 20% of the tumor, three had a history of other malignancies within the past five years, and ten individuals lacked follow-up data. Ultimately, 176 patients were included in the final analysis. Among them, 53 (30.1%) underwent MIS, while 123 (69.9%) underwent open surgery (**Table 1**). Within MIS cases, robotic-assisted laparoscopy was performed in 2 patients, and all other MIS procedures were conventional laparoscopy. The average age at diagnosis was 59.0 ± 6.9 years, with no significant difference observed between MIS and open surgery groups (59.8 ± 6.5 vs. 58.3 ± 6.9 years, P = 0.177). Similarly, there were no statistical differences in body mass index (BMI), (P = 0.341) or diabetes prevalence (P = 0.745). Although hypertension appeared more common in the MIS group (41.5%) compared to the open surgery group (28.5%), this difference was not statistically significant (P = 0.090).

**Table 1:** Clinical characteristics of patients.

Characteristics	Overall (N = 176)	Open Surgery (N = 123)	MIS (N = 53)	P value
Age, years [mean ± SD]	59.0 ± 6.9	58.3 ± 6.9	59.8 ± 6.5	0.177
BMI, kg/m² [mean ± SD]	24.1 ± 3.6	23.8 ± 3.5	24.5 ± 3.7	0.341
Hypertension				
No	119 (67.6)	88 (71.5)	31 (58.5)	0.090
Yes	57 (32.4)	35 (28.5)	22 (41.5)	
Diabetes				0.745
No	147 (83.5)	102 (82.9)	45 (84.9)	
Yes	29 (16.5)	21 (17.1)	8 (15.1)	
Stage (FIGO 2009)				0.140
I	86 (48.9)	61 (49.6)	25 (47.2)	
II	13 (7.4)	8 (6.5)	5 (9.4)	
III	53 (30.1)	33 (26.8)	20 (37.7)	
IV	24 (13.6)	21 (17.1)	3 (5.7)	
Peritoneal cytology				0.826
Negative	159 (90.3)	111 (90.2)	48 (90.6)	
Suspicious positive	5 (2.8)	3 (2.4)	2 (3.8)	
Positive	12 (6.8)	9 (7.3)	3 (5.7)	
Omentectomy				0.793
No	107 (60.8)	74 (60.2)	33 (62.3)	
Yes	69 (39.2)	49 (39.8)	20 (37.7)	
Lymphadenectomy				0.060
No	27 (15.3)	23 (18.7)	4 (7.5)	
Yes	149 (84.7)	100 (81.3)	49 (92.5)	
Cervical stromal involvement				0.440
No	132 (75.4)	90 (73.8)	42 (79.2)	
Yes	43 (24.6)	32 (26.2)	11 (20.8)	
Myometrial invasion (%)				0.447
<50	102 (58.0)	69 (56.1)	33 (62.3)	
≥50	74 (42.0)	54 (43.9)	20 (37.7)	
LVSI				0.836
No	105 (59.7)	74 (60.2)	31 (58.5)	
Yes	71 (40.3)	49 (39.8)	22 (41.5)	
Lymph node metastasis				0.902
No	124 (70.5)	87 (70.7)	37 (69.8)	
Yes	52 (29.5)	36 (29.3)	16 (30.2)	

MIS, minimally invasive surgery; BMI, Body Mass Index; FIGO, International Federation of Gynecology and Obstetrics; LVSI, Lymphovascular Space Invasion.

Regarding surgical procedures, omentectomy was performed in 39.2% of patients, with similar proportions between MIS and open groups (37.7% vs. 39.8%, P = 0.793). Lymphadenectomy was more frequently performed in the MIS group (92.5%) than in the open group (81.3%), though this difference approached but did not reach statistical significance (P = 0.060). Para-aortic lymphadenectomy was also more frequent in the MIS group (47.2% vs. 41.5%, P = 0.164), yet the difference remained non-significant. Tumor-related variables, including FIGO stage, depth of myometrial invasion, lymphovascular space invasion (LVSI), cervical stromal involvement, lymph node metastasis, and peritoneal cytology, showed no significant differences between groups. Notably, patients in the MIS group had significantly lower D-dimer levels than those in the open group (0.55 vs. 0.84 μg/mL, P = 0.033).

### Adjuvant therapy and timing

**Table 2** summarizes perioperative treatments. NACT was administered in 22/176 (12.5%) patients overall (19/123 [15.4%] after open surgery vs 3/53 [5.7%] after MIS; P = 0.072), with interval cytoreduction performed when feasible. In the overall cohort, 88.1% (155/176) of patients received adjuvant therapy, and the distribution between the MIS and open surgery groups was similar (88.7% vs. 87.8%, P = 0.870) (**Table 2**). A slightly greater proportion of patients in the MIS group received adjuvant radiotherapy compared to those in the open surgery group (66.0% vs. 59.3%, P = 0.403); however, this difference was not statistically significant. Retroperitoneal radiotherapy was administered in 39.6% of MIS cases and 27.6% of open cases (P = 0.116), while brachytherapy was given in 34.0% and 26.8%, respectively (P = 0.339), though none of these differences was statistically significant. The median vaginal radiation doses were comparable between the two surgical groups (6240 cGy for MIS vs. 5000 cGy for open surgery, P = 0.537). Adjuvant chemotherapy was administered to 84.1% of patients, with similar rates between the MIS and open surgery groups (83.0% vs. 84.6%, P = 0.799). Paclitaxel–carboplatin was the most commonly used regimen (n = 84). Subsets received cisplatin (n = 13), nedaplatin (n = 37), or lobaplatin (n = 22) as the platinum component; docetaxel substituted for paclitaxel in 18 patients, and albumin-bound paclitaxel with platinum was used in another 18 patients. The total number of chemotherapy cycles was similar between the two groups (a median of 4 cycles, P = 0.954).

**Table 2:** Comparison of adjuvant treatment between open and MIS groups.

Characteristics	Overall (N = 176)	Open surgery (N = 123)	MIS (N = 53)	P value
Neoadjuvant chemotherapy	22 (12.5)	19 (15.4)	3 (5.7)	0.072
Adjuvant therapy	155 (88.1)	108 (87.8)	47 (88.7)	0.870
Adjuvant radiotherapy	108 (61.4)	73 (59.3)	35 (66.0)	0.403
Retroperitoneal radiotherapy	55 (31.3)	34 (27.6)	21 (39.6)	0.116
Brachytherapy	51 (29.0)	33 (26.8)	18 (34.0)	0.339
Vaginal dose, cGy [median (IQR)]	5040 (4860-7410)	5000 (4800-7745)	6240 (4860-7406)	0.537
Time from Surgery to Radiotherapy, days [median (IQR)]	62 (43-110)	62 (43-110)	62 (44.5-118.5)	0.805
Adjuvant chemotherapy	148 (84.1)	104 (84.6)	44 (83.0)	0.799
Time from Surgery to chemotherapy, days [median (IQR)]	24 (18-35.25)	22 (16-32)	30 (20-44)	0.012
Chemotherapy cycles [median (IQR)]	4 (3-6)	4 (2.5-6)	4 (3-5.75)	0.954

MIS, minimally invasive surgery; IQR, interquartile range.

By surgical approach, among patients who received adjuvant chemotherapy, the median surgery-to-chemotherapy interval was 30 days (IQR 20–44) after MIS versus 22 days (IQR 16–32) after laparotomy (Mann–Whitney p=0.012). Among those who received adjuvant radiotherapy, the median surgery-to-radiotherapy interval was 62 days in both groups (MIS IQR 44.5–118.5 vs laparotomy IQR 43–110; p=0.805). In the chemotherapy-only subset, the MIS–laparotomy difference remained directionally longer for MIS (medians 26 vs 22 days) but was not statistically significant ((U = 137.5, p=0.156). Despite this difference for chemotherapy, both medians remained within our prespecified windows (chemotherapy ≤ 6 weeks; radiotherapy ≤ 12 weeks).

### Survival outcomes

**Figure 1:**
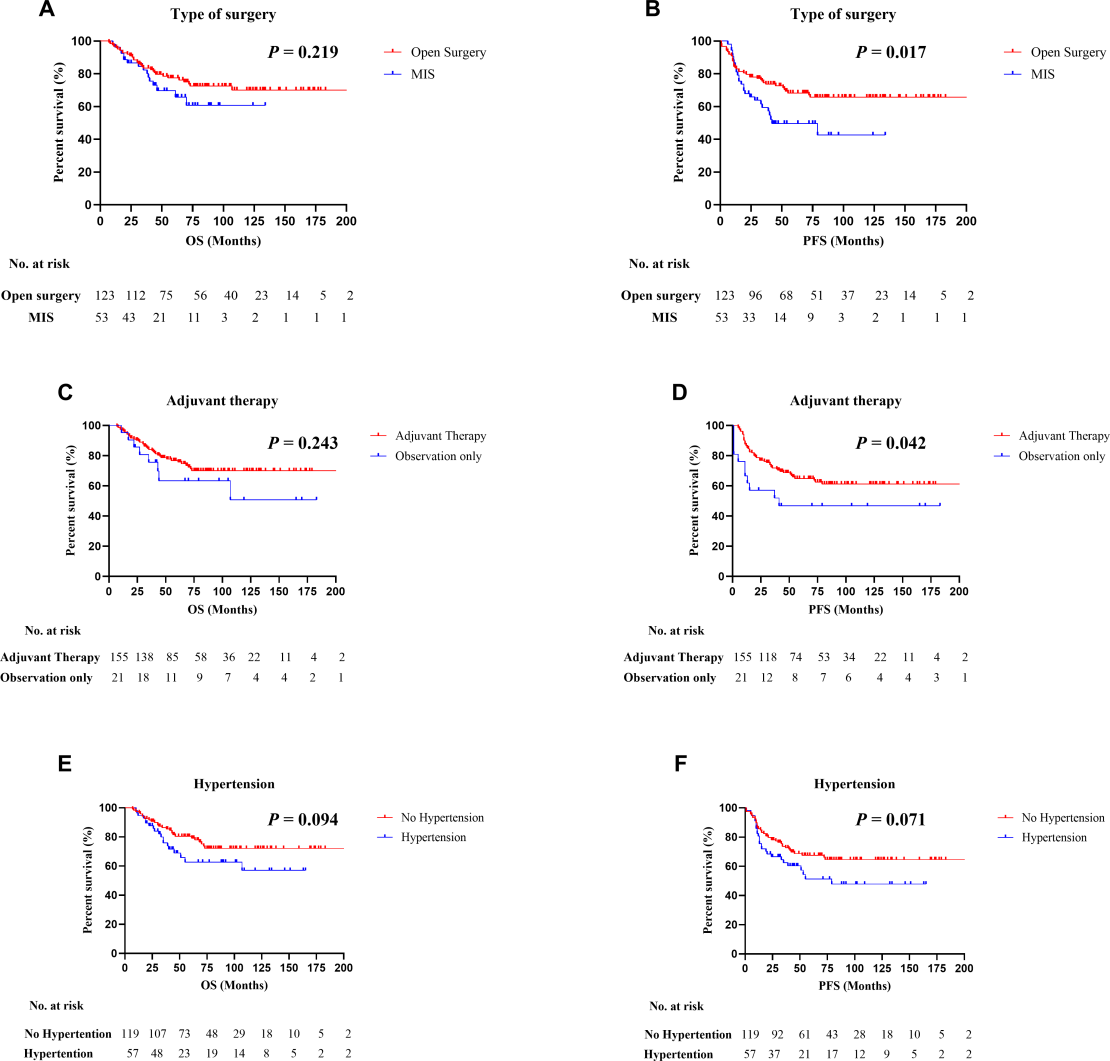
Kaplan–Meier survival curves in patients with uterine serous carcinoma (USC). **(A, B)** Comparison of progression-free survival (PFS) and overall survival (OS) between minimally invasive surgery (MIS) and open surgery groups. **(C, D)** PFS and OS stratified by receipt of adjuvant therapy. **(E, F)** PFS and OS according to hypertension status. Statistical differences were assessed using the log-rank test.

By the last follow-up (June 20, 2024), the median follow-up time was 78 months (95% CI: 68.3–87.7), while the median OS and PFS had not been reached. A total of 65 patients (36.9%) experienced disease recurrence, and 47 (26.7%) had died. The 5-year OS and PFS rates for the entire population were 75.2% and 62.7%, respectively. Patients treated with MIS demonstrated a significantly higher rate of recurrence (49.1%) compared to those undergoing open surgery (31.7%), and a lower 5-year PFS (49.7% vs. 68.3%, P = 0.017). However, no statistically significant difference in 5-year OS was observed between the MIS and open groups (69.7% vs. 77.4%, P = 0.219) (**Figure 1**).

Subgroup analyses indicated that patients with hypertension and those who did not receive adjuvant therapy tended to have poorer outcomes. Although the differences in OS and PFS were not statistically significant for hypertensive patients, they showed lower 5-year OS (62.7% vs. 80.4%) and PFS (51.3% vs. 67.6%) compared to non-hypertensive individuals. Patients who underwent adjuvant therapy exhibited better PFS outcomes than those who did not (64.9% vs. 46.8%, P = 0.042), although the OS difference between these groups was not significant (75.2% vs. 61.0%, P = 0.125).

Among 176 patients, 65 (36.9%) developed recurrence. Median PFS (time to recurrence) was 15 months (IQR 10–33) and median OS from surgery was 39 months (IQR 22–62); at the data cut-off, 47 deaths had occurred. At initial failure, allowing multiple counting for synchronous sites, the most frequent first-site categories were retroperitoneal nodes including pelvic/para-aortic (22/65, 33.8%) and pelvic (20/65, 30.8%), followed by lung (17/65, 26.2%), abdominal/peritoneal (15/65, 23.1%), distant lymph nodes (15/65, 23.1%), vaginal (12/65, 18.5%), liver (7/65, 10.8%), bone (4/65, 6.2%), and other (2/65, 3.1%). Overall, 33/65 (50.8%) had a single-site first recurrence and 31/65 (47.7%) had multi-site involvement. Post-recurrence management included chemotherapy in 37 (56.9%), radiotherapy in 15 (23.1%), salvage surgery in 6 (9.2%), targeted therapy in 12 (18.5%), and immunotherapy in 3 (4.6%); modalities were not mutually exclusive. Post-recurrence therapy was documented in 43/65 (66.2%), and 3/65 (4.6%) refused treatment. Detailed patient-level data are provided in **Supplementary Table S1**.

### Prognostic analysis of overall survival

Univariable Cox regression revealed that several clinicopathologic variables were significantly associated with OS. Specifically, worse OS was linked to advanced FIGO stage (HR = 1.698, 95% CI: 1.319–2.187, P < 0.001), myometrial invasion (HR = 2.386, 95% CI: 1.331–4.278, P = 0.004), cervical stromal involvement (HR = 2.039, 95% CI: 1.123–3.703, P = 0.019), adnexal spread (HR = 2.038, 95% CI: 1.113–3.731, P = 0.021), and lymph node metastasis (HR = 2.569, 95% CI: 1.437–4.593, P < 0.001). In contrast, surgical approach, hypertension, and receipt of adjuvant therapy did not exhibit statistically significant associations with OS in the unadjusted model (**Table 3**).

**Table 3:** Univariate and multivariate analysis of overall survival.

Characteristics	Univariable analysis			Multivariable analysis		
	HR	95% CI	P	HR	95% CI	P
FIGO stage (2009)	1.698	1.319-2.187	<0.001	1.833	1.258-2.669	0.002
Age	1.502	0.846-2.666	0.165			
Hypertension	1.638	0.913-2.937	0.098	2.057	1.111-3.811	0.022
Diabetes	1.044	0.488-2.235	0.912			
Overweight	1.329	0.738-2.395	0.343			
D-dimer	1.685	0.847-3.354	0.137			
Fibrinogen	1.732	0.958-3.131	0.069	1.556	0.813-2.979	0.182
Type of surgery	1.460	0.795-2.683	0.223	1.905	1.007-3.605	0.048
Omentectomy*	1.449	0.808-2.599	0.213			
Myometrial invasion	2.386	1.331-4.278	0.004	2.032	0.951-4.341	0.067
LVSI	1.500	0.844-2.665	0.167			
Cervical stromal involvement	2.039	1.123-3.703	0.019	1.098	0.550-2.189	0.791
Adnexal involvement	2.038	1.113-3.731	0.021	0.946	0.437-2.051	0.889
Lymph node metastasis	2.569	1.437-4.593	0.001	0.742	0.320-1.723	0.488
Adjuvant therapy	0.638	0.298-1.366	0.248	0.386	0.164-0.909	0.029

HR, Hazard Ratio; CI, Confidence Interval; FIGO, International Federation of Gynecology and Obstetrics; LVSI, Lymphovascular Space Invasion.*, “Omentectomy” denotes omental resection; cases with omental biopsy without resection were not enumerated in the table.

Multivariate Cox analysis identified four independent predictors of overall survival. Advanced FIGO stage (HR = 1.833, 95% CI: 1.258–2.669, P = 0.002), hypertension (HR = 2.057, 95% CI: 1.111–3.811, P = 0.022), and undergoing MIS (HR = 1.905, 95% CI: 1.007–3.605, P = 0.048) were each associated with reduced OS, while receipt of adjuvant therapy conferred a survival benefit (HR = 0.386, 95% CI: 0.164–0.909, P = 0.029). The previously significant variables—myometrial invasion, cervical stromal involvement, adnexal extension, and lymph node metastasis—lost statistical significance after adjustment (**Table 3**).

### Prognostic analysis of progression-free survival

In the univariable model, several factors were significantly correlated with inferior progression-free survival. These included advanced FIGO stage (HR = 1.654, 95% CI: 1.137–2.048, P < 0.001), MIS (HR = 1.819, 95% CI: 1.103–3.002, P = 0.019), myometrial invasion (HR = 2.291, 95% CI: 1.400–3.748, P = 0.001), lymphovascular space invasion (HR = 1.672, 95% CI: 1.027–2.724, P = 0.039), cervical stromal involvement (HR = 1.933, 95% CI: 1.152–3.243, P = 0.013), adnexal involvement (HR = 2.339, 95% CI: 1.405–3.894, P = 0.001), and lymph node metastasis (HR = 2.911, 95% CI: 1.780–4.761, P < 0.001). Conversely, receiving adjuvant therapy was significantly associated with improved PFS (HR = 0.519, 95% CI: 0.271–0.993, P = 0.048) (**Table 4**).

**Table 4:** Univariate and multivariate analysis of progression-free survival.

Characteristics	Univariable analysis			Multivariable analysis		
Variable	HR	95% CI	P	HR	95% CI	P
FIGO stage (2009)	1.654	1.137-2.048	<0.001	1.313	0.909-1.897	0.147
Age	1.173	0.721-1.908	0.521			
Hypertension	1.57	0.855-2.581	0.075	1.699	0.981-2.941	0.058
Diabetes	0.992	0.506-1.947	0.982			
Overweight	1.179	0.713-1.949	0.52			
D-dimer	1.673	0.940-2.976	0.08	1.538	0.829-2.854	0.172
Fibrinogen	1.502	0.913-2.473	0.109			
Type of surgery	1.819	1.103-3.002	0.019	2.293	1.313-4.005	0.004
Omentectomy*	1.238	0.754-2.032	0.398			
Myometrial invasion	2.291	1.400-3.748	0.001	1.509	0.719-3.166	0.277
LVSI	1.672	1.027-2.724	0.039	1.436	0.712-2.897	0.312
Cervical stromal involvement	1.933	1.152-3.243	0.013	0.876	0.460-1.668	0.686
Adnexal involvement	2.339	1.405-3.894	0.001	1.417	0.718-2.795	0.315
Lymph node metastasis	2.911	1.780-4.761	<0.001	1.543	0.721-3.302	0.264
Adjuvant therapy	0.519	0.271-0.993	0.048	0.278	0.129-0.598	0.001

HR, Hazard Ratio; CI, Confidence Interval; FIGO, International Federation of Gynecology and Obstetrics; LVSI, Lymphovascular Space Invasion. *, “Omentectomy” denotes omental resection; cases with omental biopsy without resection were not enumerated in the table.

Multivariate Cox regression further confirmed MIS as an independent predictor of worse PFS (HR = 2.293, 95% CI: 1.313–4.005, P = 0.004), while adjuvant therapy remained a favorable prognostic factor (HR = 0.278, 95% CI: 0.129–0.598, P = 0.001). After adjustment, the other variables—including FIGO stage, myometrial invasion, LVSI, cervical stromal involvement, adnexal spread, and lymph node metastasis—were no longer statistically significant (**Table 4**).

## Discussion

This multicenter study evaluated the prognostic significance of surgical approach and clinicopathologic variables in USC. MIS was associated with a higher risk of recurrence and shorter PFS, while the difference in OS did not reach statistical significance. In multivariable analyses, MIS remained an independent adverse factor for both PFS and OS, whereas adjuvant therapy demonstrated a favorable impact on these outcomes. Hypertension emerged as an independent adverse factor for OS.

MIS has been widely adopted for endometrial cancer because it shortens hospitalization, accelerates recovery, and reduces complications. Randomized trials (e.g., LAP2 and LACE) have shown survival comparable to laparotomy in early-stage disease (5, 7, 18); however, these trials largely enrolled low-risk endometrioid histology and included few uterine serous carcinoma (USC) cases, limiting generalizability to high-risk subtypes. The LACC trial in cervical cancer raised concerns about the oncologic safety of MIS and proposed mechanisms—use of uterine manipulators, intraoperative tumor spillage, and CO_2_pneumoperitoneum-that may also be pertinent to USC. Consistent with these concerns, we observed worse progression-free survival (PFS) with MIS. Tumor spillage has been associated with increased recurrence risk (adjusted OR 5.63, 95% CI 1.52-20.86) (19); manipulator use has been linked to conversion of peritoneal cytology from negative to positive with corresponding survival detriment (20); and a survey of gynecologic oncologists documented frequent manipulator use and nontrivial rates of perforation and spillage (21). CO_2_ pneumoperitoneum may further facilitate peritoneal dissemination: recurrence was numerically higher after total laparoscopic or robot-assisted colpotomy than after vaginal colpotomy without CO_2_ exposure (16.3% vs 5.1%, P = 0.057) (22), and instrument-related tumor seeding during laparoscopy has been reported in patients with extrauterine disease (23). Technique refinements—avoiding uterine manipulator use when feasible and otherwise minimizing uterine handling, occluding the fallopian tubes before mobilization, enclosing the specimen, and sealing the vaginal cuff prior to colpotomy-may mitigate these risks and merit prospective evaluation in USC. Notably, several USC-focused cohorts and meta-analyses have not demonstrated an adjusted survival detriment with MIS (8–11). For example, Kim et al.’s meta-analysis of mixed high-risk histologies pooled nine observational studies (n=14,628) and found no significant difference between MIS and laparotomy for recurrence (HR 0.86, 95% CI 0.71-1.05) or mortality (HR 0.86, 95% CI 0.79-0.93), with similar patterns across stage and histology subgroups (10). In contrast, in our multicenter USC-only cohort, prespecified multivariable models showed MIS independently associated with shorter PFS. The discrepancy likely reflects differences in case mix (USC-only vs mixed ‘high-risk’), stage distribution and adjuvant-therapy pathways, and residual confounding inherent to observational designs; importantly, prior pooled analyses were dominated by early-stage.

Our study identified hypertension as an independent prognostic factor for overall survival in patients with USC, consistent with previous reports (24). Although USC is categorized as a “Type II” endometrial carcinoma and exhibits weaker associations with obesity or hypertension, its prevalence is notably higher among older individuals, who more commonly have hypertension. Chronic inflammation, endothelial dysfunction, and metabolic disorders may contribute to cancer cell proliferation and invasion, ultimately affecting prognosis (25, 26). Furthermore, adjuvant therapy significantly improved PFS, reinforcing its necessity in high-risk endometrial cancer patients. A study conducted by Nasioudis et al. reported that, among patients with stage I USC, the five-year OS rate was 81.9% for those who did not receive adjuvant therapy, 85.1% for those treated with radiation alone, and 91.3% for those who underwent combined chemoradiotherapy (P < 0.001) (27). The PORTEC-3 trial further demonstrated that chemoradiotherapy significantly improved OS compared to radiation alone (71.4% vs. 52.8%, P = 0.037) in USC patients (28). While MIS was associated with shorter PFS, adjuvant therapy effectively reduced recurrence risk, underscoring the importance of comprehensive postoperative treatment (16). Although the interval to chemotherapy was longer after MIS in this cohort, this difference most likely reflects system-level scheduling and referral factors in our regional hub-and-spoke pathway (early discharge after MIS, off-site MDT clearance and oncology scheduling, inter-institutional referrals, and ancillary pathology turnaround) rather than delayed recovery. In exploratory analyses the interval itself was not significantly associated with PFS or OS; we therefore report it descriptively and avoid adjusting for this potential mediator in primary models.

This multicenter, real-world cohort from three tertiary cancer centers focuses on a rare, high-risk histology and includes a high proportion of comprehensively staged patients with long follow-up. Systematic documentation of surgical approach, adjuvant protocols and their timing, and recurrence patterns strengthens the clinical interpretability and generalizability of our findings. However, several limitations should be acknowledged. First, given its retrospective design, the study is inherently susceptible to recall and selection biases. Second, the MIS group had a relatively small sample size, potentially limiting statistical power. Additionally, variations in surgical techniques and adjuvant treatment protocols across centers, as well as the long study duration, may have introduced inconsistencies as treatment guidelines evolved over time. In addition, we observed a longer surgery-to-chemotherapy interval after MIS (median 30 vs 22 days), whereas radiotherapy timing was comparable; because both medians were within target windows and the interval was not independently associated with PFS/OS, we interpret this difference as arising from post-discharge care processes (scheduling/authorizations/inter-hospital referrals) rather than physiologic recovery. Moreover, due to resource constraints, not all USC cases were reviewed by senior pathologists, and some key laboratory parameters (e.g., CA125 levels and mismatch repair [MMR] status) were excluded due to missing data. Prospective studies with standardized techniques in uterine serous carcinoma (USC) that incorporate minimization or avoidance of uterine manipulator use, tubal occlusion before uterine mobilization and sealing of the vaginal cuff before colpotomy are warranted to evaluate whether minimally invasive surgery (MIS) preserves perioperative benefits without compromising oncologic outcomes.

## Conclusion

This study suggests that MIS is associated with an elevated risk of recurrence and a shorter PFS in patients with USC. In contrast, the application of adjuvant therapy appears effective in mitigating recurrence risk. Considering the highly aggressive behavior of USC, future investigations should prioritize the refinement of surgical approaches and postoperative management strategies to enhance clinical outcomes in this high-risk population.

## Data Availability

The original contributions presented in the study are included in the article/**Supplementary Material**. Further inquiries can be directed to the corresponding author.
